# [Retracted] MicroRNA‑505 is downregulated in human osteosarcoma and regulates cell proliferation, migration and invasion

**DOI:** 10.3892/or.2023.8582

**Published:** 2023-06-12

**Authors:** Yu-Jiang Liu, Wei Li, Feng Chang, Jian-Na Liu, Jun-Xin Lin, De-Xi Chen

Oncol Rep 39: 491–500, 2018; DOI: 10.3892/or.2017.6142

Following the publication of this paper, it was drawn to the Editor's attention by a concerned reader that the control GAPDH western blotting bands shown in Fig. 4H on p. 496 were strikingly similar to data that were submitted for publication in advance of this article in different form by different authors at different research institutes [Liu F, Bai C and Guo Z: The prognostic value of osteopontin in limited-stage small cell lung cancer patients and its mechanism. Oncotarget 8: 70084-70096, 2017]. A further independent investigation conducted in the Editorial Office revealed that other western blotting data were likely to have been shared in common, comparing between the two articles.

Owing to the fact that the contentious data in the above article had already been submitted for publication prior to the submission of this article to *Oncology Reports*, the Editor has decided that this paper should be retracted from the Journal. After having been in contact with the authors, it was admitted that the authors Feng Chang, Jian-Na Liu and Jun-Xin Lin did not initially provide their agreement to be authors on this paper; otherwise, the rest of the authors accepted the decision to retract the paper. The Editor apologizes to the readership for any inconvenience caused.

